# Autophagy and ncRNAs: Dangerous Liaisons in the Crosstalk between the Tumor and Its Microenvironment

**DOI:** 10.3390/cancers14010020

**Published:** 2021-12-21

**Authors:** Gracie Wee Ling Eng, Yilong Zheng, Dominic Wei Ting Yap, Andrea York Tiang Teo, Jit Kong Cheong

**Affiliations:** 1 Precision Medicine Programme, Yong Loo Lin School of Medicine (YLLSoM), National University of Singapore, 1E Kent Ridge Road, NUHS Tower Block Level 11, Singapore 119228, Singapore; gracie.eng@nus.edu.sg (G.W.L.E.); e0433905@u.nus.edu (Y.Z.); dominicyapweiting@u.nus.edu (D.W.T.Y.); andrea.teo@u.nus.edu (A.Y.T.T.); 2 NUS Centre for Cancer Research, National University of Singapore, 14 Medical Dr, Centre for Translational Medicine #12-01, Singapore 117599, Singapore; 3 Department of Biochemistry, YLLSoM, National University of Singapore, 8 Medical Drive, MD7 #03-09, Singapore 117597, Singapore

**Keywords:** autophagy, ncRNAs, cancer, metastasis

## Abstract

**Simple Summary:**

Tumor cells communicate with the stromal cells within the tumor microenvironment (TME) to create a conducive environment for tumor growth. One major avenue for mediating crosstalk between various cell types in the TME involves exchanges of molecular payloads in the form of extracellular vesicles/exosomes. Autophagy is a fundamental mechanism to maintain intracellular homeostasis but recent reports suggest that secretory autophagy plays an important role in promoting secretion of exosomes that are packaged with non-coding RNAs (ncRNAs) and other biomolecules from the donor cell. Uptake of exosomal autophagy-modulating ncRNAs by recipient cells may further perpetuate tumor progression.

**Abstract:**

Autophagy is a fundamental cellular homeostasis mechanism known to play multifaceted roles in the natural history of cancers over time. It has recently been shown that autophagy also mediates the crosstalk between the tumor and its microenvironment by promoting the export of molecular payloads such as non-coding RNA (ncRNAs) via LC3-dependent Extracellular Vesicle loading and secretion (LDELS). In turn, the dynamic exchange of exosomal ncRNAs regulate autophagic responses in the recipient cells within the tumor microenvironment (TME), for both tumor and stromal cells. Autophagy-dependent phenotypic changes in the recipient cells further enhance tumor growth and metastasis, through diverse biological processes, including nutrient supplementation, immune evasion, angiogenesis, and therapeutic resistance. In this review, we discuss how the feedforward autophagy-ncRNA axis orchestrates vital communications between various cell types within the TME ecosystem to promote cancer progression.

## 1. Introduction

Cancer is one of the leading causes of death worldwide, with almost 10 million cancer deaths reported in 2020 [1]. In the rapidly evolving field of cancer research, it is well documented that tumor cells do not simply exist as an isolated island of proliferative cells, but they co-exist and crosstalk with various cell types in a complex tissue microenvironment [2]. This tumor-created niche is known as the tumor microenvironment (TME). The TME evolves continuously in response to stress and aging-induced physiological functional decline. The dynamic interactions between the various cell types in the TME are mediated by exchanges of biomolecules that act harmoniously to shape the development of tumors in a highly context-dependent manner. In this review, we examine how the main players in the TME interact and influence the intracellular processes in each other to create a conducive neighborhood for growth and dissemination of cancer cells.

## 2. Major Players in the Tumor Microenvironment (TME)

The TME is made up of a variety of cell types, including tumor cells, cancer stem cells, fibroblasts, immune cells, endothelial cells, and adipocytes [3]. It is widely accepted that the interplay between the tumor cells and the stromal cells plays a key role in supporting tumor growth.

### 2.1. Tumor Cells and Cancer Stem Cells

Tumor cells are the main residents of the TME. They were once normal cells but accumulated malignant genomic alterations over time to acquire the various hallmarks of cancer: hyperproliferation, decreased growth repression and cell death, replicative immortality, increased angiogenesis, enhanced invasion and metastasis, genome instability and mutation, tumor-promoting inflammation, reprogrammed energy metabolism, and escape of immune destruction [4]. In recent decades, it has become evident that tumor cell heterogeneity exists, and studies have reported the discovery of a rare but unique population of tumor cells, coined cancer stem cells (CSCs). CSCs have subsequently been shown to be pluripotent/multipotent stem cells with self-renewal capabilities that generate proliferating differentiated cancer cells to make up the tumor bulk [5]. Given their unique growth kinetics, CSCs have been widely implicated in therapy resistance, metastasis, and relapse of tumors [6,7,8].

### 2.2. Cancer-Associated Fibroblasts

Cancer-associated fibroblasts (CAFs) represent the predominant non-hematopoietic stromal cell type in the TME [9]. Although CAFs consist of a heterogenous cell population of multiple origins [9], they share many distinct morphological and physiological features, including the expression of specific proteins such as α-smooth muscle actin and fibroblast activation protein [10]. CAFs are known to play a definite role in cancer progression, including its involvement in influencing cancer metabolism, supporting Epithelial-Mesenchymal Transition (EMT), activating angiogenesis, and modulating chemoresistance [11].

### 2.3. Mesenchymal Stem Cells

MSCs are multipotent stromal cells that have been shown to be recruited to tumors, where they serve as a source of fibroblasts and pericytes [12]. They are believed to have significant immunomodulatory effects, promote angiogenesis, and regulate tumor growth and progression [13].

### 2.4. Immune Cells

During immune surveillance, host immune cells infiltrate tumors in an attempt to halt tumor progression. Depending on the tumor type, inflammatory cells recruited to the TME vary in composition, and may include both the adaptive and innate immune cells, such as dendritic cells (DC), T-lymphocytes, B cells, macrophages, and polynuclear leukocytes [2]. Tumor-derived cytokines/chemokines, in addition to oncogenes, are believed to influence the composition and behavior of the TIME [14], leading to blockade of recruitment and/or anti-tumor functions of the immune cells through tumor–TME crosstalk.

### 2.5. Endothelial Cells

Endothelial cells (ECs) play a key role in carcinogenesis. As the tumor mass grows, the hypoxic center requires increased vasculature to aid in the delivery of nutrients and oxygen. Angiogenesis also promotes metastasis, as decreased endothelial cell–cell junctions and increased attachment of tumor cells to the ECs aids the invasion of tumor cells into the circulation. Furthermore, neovascularization renders a tumor inaccessible to chemotherapeutics [15,16].

### 2.6. Cancer-Associated Adipocytes

Adipocytes have long been hypothesized to promote tumorigenesis. Given that obesity is a risk factor for cancer, and that adipocyte mass and tissue phenotype is altered in obesity, it is highly plausible that the adipocyte–tumor cells crosstalk enhances tumor progression. Cancer-associated adipocytes (CAAs) are adipocytes activated by tumor cells to secrete proinflammatory factors known as adipokines to promote tumor growth [17].

## 3. TME Crosstalk by Autophagy-Driven Release of Exosomes

Extracellular vesicles are small, membrane-encapsulated vehicles that contain biological payloads actively secreted from cells. They are primarily categorized into two groups—exosomes and microvesicles. Exosomes are typically 30–150 nm extracellular vesicles that are secreted during the fusion of multivesicular endosomes with the cell surface [18]. Microvesicles, by comparison, are larger vesicles ranging from 50 to 1000 nm that are formed by direct outward budding of the plasma membrane [19,20]. Exosomes are reported to carry a variety of biomolecules, such as proteins, lipids, and nucleic acids. The exosomal cargoes are highly specific in nature, and are deposited into exosomes primarily through the endosomal sorting complexes required for transport (ESCRT)-dependent pathways [21]. Exosomes are then released into the extracellular space to deliver their cargoes to the recipient cells via short-range or long-distance transfer.

Exosomes have long been implicated in the communication between cancer cells and other cell types in the TME. Studies have shown that cancer or stromal cells release exosomes in order to transport lipids, proteins, and nucleic acids, including various non-coding RNAs (ncRNAs), to reprogram the recipient cells, thereby building a conducive microenvironment for cancer progression [22]. The transfer of such biomolecules has been shown to alter cell states to favor tumorigenesis by promoting several hallmarks of cancer [4], including angiogenesis, immune evasion, metastasis, and drug resistance.

### Secretory Autophagy and the TME

Autophagy is a fundamental cellular homeostasis mechanism that governs a wide variety of stress adaptation responses, including turnover of protein aggregates and organelles for nutrient recycling [23]. Autophagy is a stepwise process that involves the initiation, nucleation, and elongation of double membrane sequestering vesicles known as autophagosomes, and their fusions with lysosomes and multivesicular bodies (MVBs) for recycling of biosynthetic intermediates and exosome secretion, respectively. It is classically initiated through the PTEN/AKT/mTOR pathway, followed by nucleation by the PI3K complex that activates a signaling cascade involving several players including Beclin-1, autophagy-related proteins (ATG), and the microtubule-associated proteins 1A/1B light chain 3B (LC3). A defining feature of autophagy is the ATG4-dependent cleavage of pro-LC3 into LC3-I and its conjugation with phosphatidylethanolamine (PE) to form LC3-II for insertion into the double-membraned autophagosome membrane [24].

In mammalian cells, autophagy is canonically viewed as a lysosomal digestion process wherein specific cytoplasmic contents are delivered to lysosomes for destruction as a means of recycling biosynthetic building blocks for the maintenance of cell homeostasis [25,26,27]. Indeed, under metabolic stress, autophagy has been shown to be robustly induced in cancer cells [28,29,30]. Intriguingly, the key components of the canonical autophagy pathway have also been implicated in the secretion of cytoplasmic contents, including but not limited to cytokines [31], lysozymes [32], and extracellular vesicles [33,34]. This alternative pathway, wherein components of the classical autophagy machinery are used for the secretion instead of digestion of cytoplasmic contents, is termed secretory autophagy [35].

Central to the classical digestive autophagy and non-canonical secretory autophagy pathways is LC3. In classical digestive autophagy, LC3 identifies and transports proteins to the autophagosome for subsequent digestion in the lysosome [36]. In secretory autophagy, the lipidated isoform of LC3, LC3-II, identifies RNA and proteins [35,37] to recruit them to precursors of exosomes for secretion into the extracellular environment [38]. Specifically, LC3-II is required for the loading of specific RNA-binding proteins and their ncRNA cargoes into exosomes via the secretory autophagy pathway termed LC3-dependent EV loading and secretion (LDELS) [35]. RNA-sequencing of the exosomal RNAs revealed that 76% of LDELS-regulated exosomal small ncRNAs are small nucleolar RNAs (snoRNAs) or fragments of snoRNAs, followed by microRNA (miRNA), transfer RNA (tRNA), and small nuclear RNA (snRNA) [35]. Exosomal secretion of LC3-II and RBP requires neutral sphingomyelinase 2 (nSMase2) and LC3-dependent recruitment of factor associated with nSMase2 activity (FAN) [35]. Briefly, LC3 recruits FAN via a conserved LC3-interaction region [39]. FAN then stimulates nSMase2-dependent production of ceramide to facilitate intraluminal budding and the formation of intraluminal vesicles (or endosomes), prior to their fusion with the cell membrane and eventual release in the form of exosomes [39].

**Figure 1:**
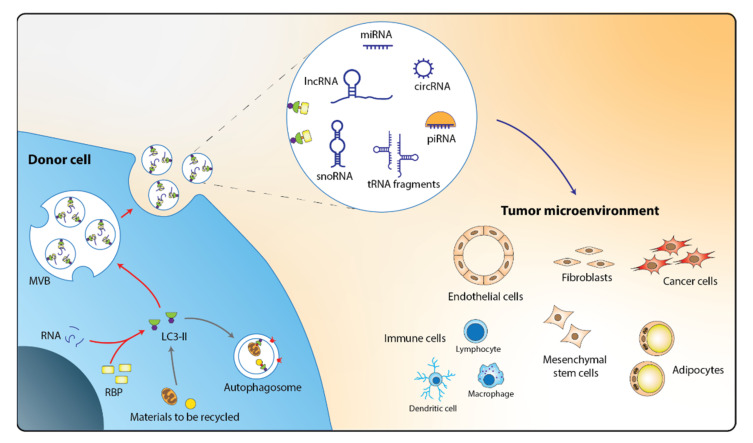
Autophagy mediate crosstalk in the TME via export of ncRNAs into exosomes. In the secretory autophagy LDELS pathway, LC3-II recruits various RBP-bound ncRNAs into exosomes prior to their export from the donor cells. These molecular cargo-loaded exosomes are taken up by recipient cells in the TME. LDELS: LC3-dependent EV loading and secretion; MVB: multivesicular bodies; RBP: RNA-binding protein.

This was a very recent seminal discovery by the Debnath laboratory, because only the ESCRT-dependent pathway was previously known to drive exosomal secretion [39,40]. Although nSMase was previously implicated in the exosomal secretion of miRNAs [41], much remained unknown about the regulation of its activity and its specificity of cargo selection until the landmark study by Leidal and coworkers [35]. Notably, this non-canonical exosomal secretion pathway appears to exist in both cancer and non-cancer cells, indicating that the crosstalk between tumor and TME may be mediated, in part, by autophagy-driven release of exosomes (Figure 1). This corroborates other studies that demonstrated intercellular exchanges of exosomal ncRNAs between tumor cells and the TME [42,43].

## 4. Exosomal ncRNAs Modulate Autophagy in TME Crosstalk

ncRNAs were first detected in exosomes and have been postulated to serve as second messengers to mediate cell-to-cell communication [37]. ncRNAs make up more than 90% of all RNAs that are transcribed from the human genome, and by definition lack protein-encoding information [44]. ncRNAs are broadly categorized into two major groups based on sequence length, in which long ncRNAs are typically longer than 200 nucleotides and small ncRNAs frequently comprise 200 or fewer nucleotides [45]. They have been implicated in diverse molecular processes, including the regulation of gene expression, post-translational modifications, and protein translation. As dysregulation of ncRNA expression is frequently linked to human diseases such as cancer, they have been postulated to serve as good biomarkers, therapeutic targets, and even as therapeutic agents [44,46].

The packaging of ncRNAs into exosomes is largely dependent on their protein binding partners [47] and their export from donor cells is mediated by the ESCRT- or LDELS-dependent pathways. Exosomal ncRNAs have been shown to modulate a multitude of cellular processes such as epithelial-mesenchymal transition, angiogenesis, establishment of pre-metastatic niche and metastasis, immune response, and therapeutic resistance [19]. Although the role of exosomal ncRNAs in the crosstalk between tumor and the TME has been comprehensively discussed by others [19,22,48,49,50,51,52,53], we specifically focus on how autophagy-modulatory ncRNAs may modulate the TME such that it becomes conducive to tumor growth. We summarize, in Table 1, notable examples of exosomal ncRNAs that have been reported to be (1) secreted by tumor cells to regulate the other cell types in the TME and (2) reciprocally secreted by other cell types in the TME to modulate tumor cell behavior.

### 4.1. miRNAs

miRNAs are small ncRNAs that are 19–25 nucleotides long. They have been largely shown to downregulate gene expression via the RNA-induced silencing complex (RISC). Notably, recent studies demonstrated that gene expression could also be upregulated by miRNAs in specific contexts, such as assembling the ribosomal complex at 5′ UTR of messenger RNA (mRNA) transcripts to promote protein translation [71]. Given that miRNAs are actively secreted from cells in exosomes or protein/lipid-bound forms, they are extremely stable in the circulation and, hence, are clinically relevant biomarker candidates [72].

To date, miRNAs are the most extensively studied group of exosomal ncRNAs and their roles in cancer were comprehensively reviewed by Sun and coworkers [50]. Exosomal miRNAs affect multiple cell types in the TME, including cancer cells, fibroblasts, endothelial cells, and immune cells. Although uptake of exosomal miRNAs leads to pleiotropic effects in the recipient cells in the TME, tumor growth is invariably enhanced and may metastasize at a later stage. For instance, MBA-MD-231 and MCF7 breast cancer (BC) cells have been found to secrete miR-126 to activate the AMP-activated protein kinase (AMPK)/autophagy pathway in co-cultured mature 3T3-L1 white adipocytes. The BC-secreted miR-126 led to browning of the co-cultured white adipocytes and their increased catabolism, which in turn promoted the transfer of adipocyte-derived metabolites to the BC cells to enhance their growth rate. Intriguingly, AMPK phosphorylation appeared to increase in these BC cells, suggesting that autophagy may be involved in the export of BC-specific factors such as miR-126 to the TME [54]. miR-1910-3p was also found in exosomes from BC cells, and uptake of these exosomes by normal mammary epithelial cells lead to downregulation of MTMR3, resulting in activation of NF_k_B signaling and downstream autophagy. Consequently, these epithelial cells exhibit increased proliferation and migration [55]. Conversely, MCF10a mammary epithelial cells have been shown to secrete exosomal miR-567 that is taken up by trastuzumab-resistant SKBR-3 and BT474 BC cells. miR-567 downregulates ATG5 and suppresses autophagy in the BC cells, leading to their increased sensitivity to trastuzumab treatment [62]. In addition, exosomal miR-1434 from TP53-inactivated colorectal cancer (CRC) cells have been shown to be internalized by normal fibroblasts to suppress autophagy by targeting intracellular ATG2B, which led to the activation of the fibroblasts and induction of fibroblast-mediated cancer cell proliferation [56].

In addition to augmenting tumor growth, exosomal miRNAs also play important but under-appreciated roles in inducing therapy resistance and side effects. The transfer of exosomal miR-425-3p from cisplatin-treated non-small cell lung cancer (NSCLC) cells to their cisplatin-naïve counterparts decreased the sensitivity of the recipient cells to subsequent cisplatin treatment. This is, in part, attributed to the activation of autophagy in the recipient cells by miR-425-3p-dependent targeting of AKT1 [63]. Similarly, drug-resistant hepatocellular carcinoma (HCC) cells secrete miR-32-5p in exosomes that are taken up by drug-sensitive HCC cells to activate autophagy by downregulating PTEN. This confers multi-drug resistance in the drug-sensitive HCC cells and enhances their proliferation and migration [64]. On the contrary, downregulation of miR-30a has been proposed to be the leading cause of cisplatin resistance in oral squamous cell carcinoma (OSCC). Overexpression of miR-30a restored cisplatin sensitivity to cisplatin-resistant OSCC cells [65]. Exosome-mediated transfer of miR-30a into the non-transfected cisplatin-resistant OSCC cells led to the downregulation of Beclin-1 and suppression of autophagy, thus re-sensitizing the OSCC cells to cisplatin treatment [65].

### 4.2. lncRNA

Long non-coding RNAs (lncRNAs) represent another group of RNA species that are commonly found in exosomes. They have sequence lengths that range from 200 bp to 10 kb and are functionally diverse. lncRNAs can be categorized based on (1) how they are synthesized (whether they are transcribed from intergenic or intronic sites of the genome in sense or anti-sense direction), and (2) their functions (cis-acting lncRNA functions at sites where they are transcribed or trans-acting lncRNAs function at sites different from where they are transcribed). In general, IncRNAs regulate gene expression by direct interaction with gene regulatory elements or recruitment of regulatory protein effectors to the site of action [51].

The opposing roles of lncRNAs in various forms of cancers have been well documented. A growing number of studies have demonstrated that lncRNAs drive chemoresistance, metastasis, and proliferation of cancer cells, possibly via exerting their effects on the different stages of autophagy (initiation, phagophore nucleation, elongation, closure, and fusion) [73,74]. Notably, lncRNAs are frequently found to be secreted in exosomes, promoting the crosstalk between the different cell types within the TME [51].

Exosomes from CD90+ liver cancer cells have been shown to be enriched in lncRNA H19, and uptake by human microvascular vein endothelial cells (HUVECs) leads to increased angiogenesis [59]. lncRNA H19 was subsequently shown to induce hypoxic injury by upregulating autophagy via the PI3K/Akt/mTOR pathway in HCC [75]. Exosomal transfer of lncRNA H19 from Erlotinib-resistant NSCLC cells to Erlotinib-sensitive cells conferred resistance to the recipient cells. lncRNA H19 was shown to downregulate miR-615-3p, which is a regulator of ATG7 [68]. Similarly, lncRNA AGAP2-AS1 has been found to be disseminated in exosomes produced by Trastuzumab-resistant BC cells, and subsequently internalized by HER2+ BC cells to promote trastuzumab resistance. lncRNA AGAP2-AS1 enhance ATG10 transcription, thereby activating autophagy in BC cells [69]. MALAT1 is another well-studied lncRNA that is frequently found in NSCLC-derived exosomes [76]. Notably, higher serum exosomal MALAT1 is associated with advanced stages of NSCLC [77]. MALAT1 has also been shown to promote dendritic cell autophagy in mouse models, leading to decreased phagocytosis and inflammatory response [60]. In osteosarcoma, transfer of lncRNA OIP5-AS1 sponges miR-153, thereby activating autophagy via ATG5 expression. This leads to enhanced cell migration, invasion, and angiogenesis [67].

Exosomal lncRNAs have also been reported to suppress autophagic functions in recipient cells. For instance, FJ22447 (also known as lncRNA-CAF), which was isolated from OSCC exosomes, has been shown to prevent autophagy-dependent degradation of Interleukin-33 (IL-33), leading to a cancer-associated fibroblast phenotype that enhanced tumor growth and proliferation [57]. SNHG9 enriched in the exosomes of papillary thyroid cancer (PTC) cells inhibits autophagy via the YBOX3/p21/p38 MAPK axis in recipient epithelial cells, leading to increased apoptosis [58]. LINC00470 has been found to be enriched in the serum of glioma patients. It was shown to inhibit autophagy by sequestering miR-589-3p and inducing derepression of WEE1 expression, leading to increased proliferation in U251 and SWO-38 glioma cells [66].

### 4.3. circRNA

The influence of exosomal circular RNAs (circRNAs) on autophagy and the consequent effects on cancer pathogenesis have been described but not as extensively as those of exosomal miRNAs and lncRNAs [52,53,78]. circRNAs are circularized fragments of RNAs. They function mainly as miRNA sponges, although they have also been shown to bind to proteins, and may be translated into proteins [78]. Exosomal circRNAs were first reported in 2015 [79] to predominantly be found in serum-derived exosomes of healthy donors. circRNAs have been implicated in tumor proliferation, metastasis, and drug resistance [80,81]. Circ-NRIP1 has been shown to act as a miR-149-5p sponge, thereby suppressing autophagy through the AKT1/mTOR pathway, promoting gastric cancer (GC) cell proliferation and altered energy metabolism [70]. Multiple myeloma cells secrete circ-G042080 to increase autophagy in cardiomyocytes via miR-4268/TLR4 axis, leading to autophagy-dependent cell death [61].

### 4.4. snoRNAs

Small nucleolar RNAs (snoRNAs) are a class of ncRNAs primarily located in the nucleolus. They play essential roles as guide RNAs in post-transcriptional modification of target RNAs, but numerous reports have revealed alternative roles for snoRNAs [82]. snoRNAs can be processed into snoRNA-derived RNAs (sdRNAs) which were shown to perform miRNA-like gene regulatory activity. snoRNAs and their derivatives have been found in plasma exosomes, and were demonstrated to be potential biomarkers in cancer [83,84]. SNORD28 (also known as U28), a snoRNA detected in LDELS-regulated exosomes [35], has been found to be the precursor of sno-miR-28 that regulates the expression of TAF9B [85]. TAF9B stabilizes p53, a known regulator of autophagy [86]. It is therefore plausible that exosomal sno-miR-28 may modulate autophagy in the tumor TME.

### 4.5. Other ncRNAs

ncRNAs such as piRNA [87,88] and tRNA fragments [89,90] have also been detected in plasma exosomes by RNA sequencing. Although they were found to be potential biomarkers, their effects on the TME and autophagy remain unclear.

## 5. Autophagy in the TME Stromal Cells Promote Tumorigenesis

**Figure 2:**
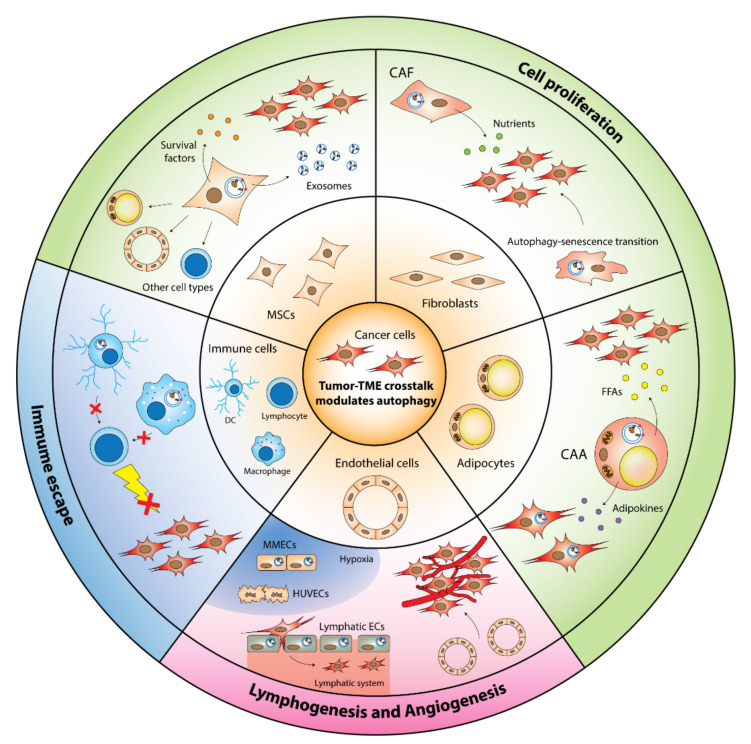
Autophagy in TME cells drives tumorigenesis. The activation or suppression of autophagy in the TME cells can reprogram their gene networks to modify the cells’ phenotypes, leading to disease progression through enhanced cell proliferation, modified lymphogenesis or angiogenesis, and immune escape. CAA: Cancer-associated adipocyte; CAF: Cancer-associated fibroblast; DC: Dendritic cell; EC: Endothelial cell; FFA: Free fatty acid; HUVEC: Human umbilical vein endothelial cell; MMEC: multiple-myeloma endothelial cell; MSC: Mesenchymal stem cell.

The roles of autophagy in tumor cells in helping to create a permissive pro-growth TME have been discussed in other reviews [91,92,93,94]. However, emerging evidence has demonstrated that autophagy in the TME stromal cells can also fuel tumor progression in a feed-forward manner. Here, we dissect the distinct roles of autophagy in the various TME stromal cell types in driving tumor development (Figure 2).

### 5.1. CAFs

Autophagy in CAFs promotes tumor growth by supplying paracrine-produced nutrients to the cancer cells [95]. Multiple studies have demonstrated the potentiating effect of autophagy in CAFs on various cancers, including colorectal cancer, breast cancer, and head and neck cancers [96,97,98,99]. This is attributed to the autophagic destruction of mitochondria within the CAF because of oxidative stress exerted by cancer cells. CAFs are then forced to undergo aerobic glycolysis and produce energy-rich nutrients (such as lactate and ketones), which cancer cells rely on to grow and proliferate [100,101]. Hypoxia in the TME has been found to induce the autophagic degradation of stromal caveolin-1 (Cav-1) in stromal fibroblasts [102], along with the concomitant upregulation of well-established autophagy markers such as HIF-1α and NFκB. It was further demonstrated that activation of HIF-1α and NFκB through oxidative stress in fibroblasts triggers the autophagic degradation of Cav-1. The loss of Cav-1 in stromal fibroblasts, in turn, reduced adjacent cancer cell apoptosis [103].

In addition, several studies suggest that cellular senescence and autophagy may participate in the same metabolic pathway, known as the autophagy-senescence transition [104,105,106,107]. Cellular senescence refers to the phenomenon where cells reach a state of stable and long-term loss of proliferative capacity, while retaining normal metabolic activity and viability [108]. Senescence has been classically viewed as a tumor-suppressive mechanism. However, senescent cells in the TME, especially fibroblasts, have increasingly been shown to promote tumorigenesis, via the secretion of pro-inflammatory cytokines, chemokines, and growth factors, which are collectively known as the senescence-associated secretory phenotype (SASP) [108,109]. Autophagic-senescent fibroblasts can also stimulate mitochondrial metabolism in adjacent cancer cells, leading to induction of metastasis [105]. Alternatively, CAFs have also been shown to support tumor growth via (1) autophagy-induced EMT, (2) promoting stemness, and (3) reducing drug sensitivity of tumor cells [110]. As autophagy appears to exert demonstrate opposing effects of autophagy on cellular senescence, we envisage that the complex relationship between senescence and autophagy is likely to be dependent on the cell- and tissue-specific context. Conjectures to account for this apparent discrepancy have also been described elegantly elsewhere [111,112].

### 5.2. MSCs

Like CAFs, autophagy modulation in mesenchymal stem cells (MSCs) is also associated with tumorigenesis. In vitro studies have demonstrated that serum deprivation led to an increase in autophagy in MSCs, promoting survival and supporting adjacent tumor cell growth by secreting paracrine factors [113]. MSCs were reported to undergo mitophagy during oxidative stress and package mitochondria into MVBs for extracellular transfer [114]. Interestingly, the resultant exosomes also contained miRNAs, which may have been loaded via the previously unknown LDELS. Exosomes secreted by MSCs have been shown to affect the development of multiple cancer types such as BC and osteosarcoma [115,116,117].

Autophagy in MSCs also influences the composition and function of other stromal cells in the TME. Activation of autophagy by NOTCH inhibition in human bone marrow-derived MSCs (BM-MSCs) led to adipogenic differentiation [118], which may play a role in the provision of nutrients for the tumor cells. Activation of autophagy in MSCs resulted in enhanced recruitment of co-cultured CD4+ T-cells, and modulation of the ratio of the T cell population [119]. Interestingly, autophagy led to an increase in anti-inflammatory regulatory T cells while decreasing pro-inflammatory Th1 helper cells [119], providing a favorable environment for tumorigenesis. Hypoxia in the TME [120] was shown to induce autophagy in BM-MSCs though the ERK1/2 pathway [121]. Autophagy induction in MSCs induced VEGF secretion and vascularization in the skin [122], a mechanism that can be hijacked by tumor cells to benefit the TME.

### 5.3. Immune Cells

The role of autophagy in the immune response is well documented [123,124]. Autophagy is implicated in both innate and adaptive immunity. For instance, multiple Toll-like receptors (innate immune receptors) have been reported to stimulate autophagy to enhance the host response [125,126,127]. Autophagy also provides fuel in the form of ATPs for anti-tumor T lymphocytes to engage and activate antigen presenting cells to trigger the adaptive immune response [128,129].

Several studies have shown that autophagy impairs antigen presentation, thereby affecting the anti-tumor immune response. In pancreatic ductal adenocarcinoma, MHC-1 molecules are selectively targeted for lysosomal degradation via an autophagy-dependent mechanism involving autophagy cargo receptor NBR1. Inhibition of autophagy conversely led to an enhanced anti-tumor response, as the presence of more surface MHC-1 molecules improved antigen presentation and enhanced anti-tumor T cell responses [130]. Production of TIM-4, expressed on tumor-associated myeloid cells such as tumor-associated macrophages and dendritic cells, has been shown to be induced by damage-associated molecular patterns (DAMPs) released from chemotherapy-damaged tumor cells. TIM-4, in turn, activated autophagy-mediated degradation of ingested tumor cells, thereby reducing antigen presentation and impairing CD8+ cytotoxic T-lymphocyte responses. TIM-4 blockade enhances antigen cross-presentation and anti-tumor response [131]. Similarly, several immune components of the TME can also induce autophagy. At high effector-to-target ratios, human peripheral blood lymphocytes also demonstrated an ability to promote autophagy in TME cells from several human tumors, with natural killer cells acting as a primary mediator of this process [132]. Moreover, autophagy is associated with the secretory functions of innate immune cells, such as cytokine release, degranulation, and exosome secretion, and autophagy deficiency led to deregulated immune function [133]. Conversely, the immune cells can be the recipients of exosomal ncRNA transfer from a subset of cancers. For instance, Epstein–Barr Virus (EBV)-infected cancer cells have been shown to secrete viral miRNAs to modulate gene expression in immune cells, promoting immune escape to cancer cells via inhibition of CD4+ T-cell response [134,135].

### 5.4. ECs

In mice, EC-specific ATG5 knockdown led to an increased number of immature blood vessels with abnormal EC lining [136]. Beclin1 deletion in ECs enhances their proliferation, migration, tube formation, and hypoxia-induced angiogenesis [137]. Taken together, these studies suggest that autophagy inhibits normal angiogenesis in ECs. In contrast, multiple myeloma endothelial cells (MMECs) exhibit higher basal autophagy than human umbilical vein endothelial cells (HUVECs), and are protected from hypoxia-induced cell death [138]. In lymphatic ECs, Paclitaxel exposure induces autophagy in ECs, disrupting the endothelial barrier and promoting nodal metastasis [139].

Although these reports appear to be contradictory, the discrepancy in observations may be partially due to differences in the models used (in vivo vs. in vitro). It is noteworthy that ATG5 and Beclin1 are known to participate in alternative cellular pathways independent of autophagy, which may potentially convolute our assessment of the real effect of autophagy on ECs in the TME. Hence, additional studies are warranted to determine the exact role(s) of autophagy in ECs in tumor angiogenesis and other pro-tumor effects.

### 5.5. CAAs

Autophagy is known to regulate lipid metabolism in adipocytes by a process known as lipophagy, in which the autophagy machinery breaks down lipid droplets into free fatty acids (FFAs) [140]. FFAs are then secreted from adipocytes to provide energy to surrounding cells during starvation. It is perhaps not surprising that autophagy can be upregulated in CAAs to fuel tumorigenesis and metastasis [54]. CAAs have also been shown to promote tumor proliferation, growth, and treatment resistance by activating autophagy in tumor cells such as colon cancer [141], multiple myeloma [142], and breast cancer [143].

## 6. Conclusions and Future Perspectives

Autophagy plays a vital role in the crosstalk between various cell types of the tumor, stroma, and immune system within the TME. We summarized the multifaceted roles of autophagy in mediating exchanges of exosomal ncRNA effectors across different cell types. The autophagy-dependent LDEL secretory pathway loads ncRNAs into exosomes for export into the extracellular space to mediate cell-to-cell communications. Exosomal ncRNAs are secreted from the various cell types in the TME and are taken up by recipient cells to regulate cellular processes (including autophagy) in a highly specific but elusive manner to promote or suppress cancer growth. Hence, it will be critical to delineate the composition of exosomes and their biological payloads (including ncRNAs) from each of these cell types in the TME to fully understand the complexity of these cellular conversations. We also described how the stromal cells respond to the changes in autophagic flux and contribute to further disease progression.

In conclusion, we envisage that ongoing efforts to specifically target second messengers in exosomes or selective delivery of autophagy inhibitors or autophagy-modulatory ncRNAs to specific cell types in the TME will contribute to our armamentarium against cancer. Most RNA-based therapeutics in clinical trials target mRNAs. Although an increasing number of ncRNA therapeutics are being evaluated in clinical trials (Table 2), none of these appear to target autophagy. As ncRNAs deregulated in cancers typically affect multiple pathways, focusing on a single pathway may be less effective than targeting the ncRNA itself. Given that autophagy is also necessary for the homeostasis of non-cancer cells, therapeutics targeting autophagy should be specific for cells in the TME, to avoid potentially undesirable off-target effects on cells located outside of the TME. ncRNAs have the potential to achieve this specific targeting of cancer cells. For example, many exosomal ncRNAs secreted by non-cancer cells in the TME do not have significant effects on the donor cells. However, they confer drug resistance to the recipient cancer cells, through modulating autophagy (Table 1). By administering agents that target these TME-derived ncRNAs concurrently with conventional chemotherapeutics, it would theoretically be possible to pre-emptively overcome therapeutic resistance. In the future, it would be interesting to explore the efficacy of other exosomal packaging systems to deliver nucleotide-based therapeutics to target cells in a stable and specific manner. Indeed, MSC-derived and red blood cell-derived exosomes have been shown to be promising delivery systems [144,145].

However, we suggest that therapeutic resistance in cancer is fundamentally inevitable. Even if we do eventually find effective agents that target the autophagy-ncRNA axis, the presence of intra-tumoral heterogeneity means that there may be cancer cells that are innately resistant to these ncRNA-targeting therapies. The presence of inter-tumoral heterogeneity also means that therapies that work in one patient may not necessarily work in another. Cancer is an incredibly complex disease, and we still have much to learn about it.

Nevertheless, by harnessing our knowledge of exosomal ncRNAs that mediate the conversation between the tumor and its microenvironment, we can seize the opportunity to selectively target important pro-tumorigenic signals to control tumor growth more effectively.

## Figures and Tables

**Table 1:** Exosomal ncRNAs modulating autophagy in tumor–TME crosstalk.

ncRNA	Target	Effect on Autophagy	Donor Cells	Recipient Cells	Cancer	Reference
Tumor to TME						
miR-126	AMPK	Activated	MBA-MD-231; MCF7	Mature L-313 adipocytes	BC	[54]
miR-1910-3p	MTMR3	Activated	MBA-MD-231; MCF7	MCF10a epithelial cells	BC	[55]
miR-1434	ATG2B	Suppressed	TP-53 inactivated CRC cells	CCD-18Co fibroblasts	CRC	[56]
lncRNA FLJ22447 (lncRNA-CAF)	IL-33	Suppressed	HSC3 cells	OSCC-derived normal fibroblasts	OSCC	[57]
lncRNA SNHG9	YBOX3	Suppressed	TPC-1; K-1	Nthy-ori-3 thyroid epithelial cells	PTC	[58]
lncRNA H19	Undetermined	Activated	CD90+ Huh7 cells	HUVECs	HCC	[59]
MALAT1	Undetermined	Activated	LLC cells	Dendritic cells	NSCLC	[60]
circ-G042080	miR-4268	Activated	U266 cells	H9C2 cardiomyocytes	MM	[61]
TME to tumor						
miR-567	ATG5	Suppressed	MCF10a	Trastuzumab-resistant BC cells	BC	[62]
miR-425-3p	AKT1	Activated	Cisplatin-treated A549	Cisplatin-naïve A549	NSCLC	[63]
miR-32-5p	PTEN	Activated	Multi-drug resistant Bel/5-FU	Drug sensitive Bel7402	HCC	[64]
miR-30a	Beclin-1	Suppressed	Cisplatin-resistant OSCC cells expressing miR-30a-mimic	Cisplatin-resistant OSCC cells	OSCC	[65]
lncRNA LINC00470	miR-580-3p	Suppressed	circulating serum exosome	U251 and SWO-38 cells	Glioma	[66]
lncRNA OIP5-AS1	miR-153	Activated	Osteosarcoma cells	Osteosarcoma cells	Osteosarcoma	[67]
lncRNA H19	miR-615-3p	Undetermined	Erlotinib-resistant NSCLC cells	Erlotinib-sensitive NSCLC cells	NSCLC	[68]
lncRNA AGAP2-AS1	ATG10	Activated	Trastuzumab-resistant SKBR-3	Trastuzumab-sensitive BC cells	BC	[69]
CircNRIP1	miR-149-5p	Suppressed	Gastric cancer cell lines	Gastric cancer cell lines	GC	[70]

BC: Breast cancer; CRC: Colorectal cancer; EC: endothelial cells; GC: Gastric cancer; HCC: Hepatocellular carcinoma; HUVECs: Human umbilical vein endothelial cells; MM: Multiple myeloma; NSCLC: Non-small cell lung cancer; PTC: papillary thyroid cancer; OSCC: Oral squamous cell carcinoma.

**Table 2:** Examples of ncRNA therapeutics for cancer currently in clinical trials.

Therapeutic ncRNA	Type	Modification and Delivery	Route of Administration	Disease	Target Gene and Pathway	Phase	Identifier
Cotsiranib (STP705)	siRNA	PNP-enhanced delivery	Intratumoral injection	Squamous Cell Carcinoma; Cutaneous Squamous Cell Carcinoma in situ (isSCC, Bowen’s disease); Basal cell carcinoma; Cholangiocarcinoma; HCC; Liver metastasis	TGF-β1 mRNA COX-2 mRNA	I/II	NCT04844983 NCT04293679 NCT04669808 NCT04676633
EGFR antisense DNA (EGFR AS)	ASO	Antisense DNA in a modified pNGVL vector	Intratumoral injection	Head and neck cancer	EGFR mRNA	I/II	NCT01592721
IONIS-AR-2.5Rx (ARRx; AZD5312)	ASO	PS 2′-cEt	Intravenous	Castration resistant prostate cancer	Androgen Receptor mRNA	I/II	NCT03300505
Danvatirsen (AZD9150)	ASO	PS 2′-cEt	Intravenous	NSCLC; CRC; HCC	STAT3 mRNA	I/II	NCT02983578 NCT03334617 NCT01839604
BP1001BP1001-A	ASO	Liposome- incorporated antisense DNA	Intravenous	AMLsolid tumor	Grb2 mRNA	III	NCT02781883 NCT04196257
siG12D-LODER	siRNA	Miniature biodegradable polymeric matrix	Intratumoral injection	Pancreatic cancer	Kras G12D mRNA	I/II	NCT01676259 NCT01188785
INT-1B3	miRNA	Nanoparticle formulated	Intravenous	solid tumor	miR-193a-3p targetome	I	NCT04675996
iExosomes	siRNA	MSC derived exosomes	Intravenous	Pancreatic cancer	Kras G12D mRNA	I	NCT03608631
EphA2-siRNA	siRNA	DOPC- encapsulated in liposome	Intravenous	Solid tumors	EphA2 mRNA	I	NCT01591356
CpG-STAT3 siRNA CAS3/SS3	siRNA	CpG-ODN linked siRNA	Intratumoral injection	B-cell NHL	STAT3 mRNA	I	NCT04995536
TASO-001 (ATB-301)	ASO	S-ODN	Intravenous	solid tumor	TGF- β2 mRNA	I	NCT04862767
BP1002	ASO	Liposome- incorporated antisense DNA	Intravenous	Lymphoid malignancies	L-Bcl-2 mRNA	I	NCT04072458
AZD8701	ASO	PS 2′-cEt	Intravenous	Solid tumors	FoxP3 mRNA	I	NCT04504669

ASO: antisense oligonucleotide; CpG-ODN: CpG oligodeoxynucleotides; DOPC: 1,2-Dioleoyl-sn-glycero-3-phosphocholine; ODN: oligodeoxynucleotide; PNP: Polypeptide nanoparticle; PS 2′-cEt: phosphorothioate 2′-constrained ethyl [146]; S-ODN: phosphorothioate-modified antisense oligodeoxynucleotide.
